# Exploring the dynamics of gut microbiota, antibiotic resistance, and chemotherapy impact in acute leukemia patients: A comprehensive metagenomic analysis

**DOI:** 10.1080/21505594.2024.2428843

**Published:** 2024-12-02

**Authors:** Ying Luo, Taha Majid Mahmood Sheikh, Xin Li, YuMeng Yuan, Fen Yao, Meimei Wang, Xiaoling Guo, Jilong Wu, Muhammad Shafiq, Qingdong Xie, Xiaoyang Jiao

**Affiliations:** a Department of Laboratory Medicine, The Second Affiliated Hospital of Shantou University Medical College, Shantou, China; b Department of Cell Biology and Genetics, Shantou University Medical College, Shantou, China; c Research Institute of Clinical Pharmacy, Shantou University Medical College, Shantou, China

**Keywords:** Acute leukemia, metagenomics, chemotherapy, gut microbiota, antibiotic resistance genes (ARGs)

## Abstract

Leukemia poses significant challenges to its treatment, and understanding its complex pathogenesis is crucial. This study used metagenomic sequencing to investigate the interplay between chemotherapy, gut microbiota, and antibiotic resistance in patients with acute leukemia (AL). Pre- and post-chemotherapy stool samples from patients revealed alterations in microbial richness, taxa, and antibiotic resistance genes (ARGs). The analysis revealed a decreased alpha diversity, increased dispersion in post-chemotherapy samples, and changes in the abundance of specific bacteria. Key bacteria such as *Enterococcus, Klebsiella*, and *Escherichia coli have* been identified as prevalent ARG carriers. Correlation analysis between gut microbiota and blood indicators revealed potential links between microbial species and inflammatory biomarkers, including C-reactive protein (CRP) and adenosine deaminase (ADA). This study investigated the impact of antibiotic dosage on microbiota and ARGs, revealing networks connecting co-occurring ARGs with microbial species (179 nodes, 206 edges), and networks associated with ARGs and antibiotic dosages (50 nodes, 50 edges). Antibiotics such as cephamycin and sulfonamide led to multidrug-resistant *Klebsiella* colonization. Our analyses revealed distinct microbial profiles with *Salmonella enterica* elevated post-chemotherapy in NF patients and *Akkermansia muciniphila* elevated pre-chemotherapy. These microbial signatures could inform strategies to modulate the gut microbiome, potentially mitigating the risk of neutropenic fever in patients undergoing chemotherapy. Finally, a comprehensive analysis of KEGG modules shed light on disrupted metabolic pathways after chemotherapy, providing insights into potential targets for managing side effects. Overall, this study revealed intricate relationships between gut microbiota, chemotherapy, and antibiotic resistance, providing new insights into improving therapy and enhancing patient outcomes.

## Introduction

Leukemia, particularly Acute Leukemia (AL), is a malignant disease that leads to clonal expansion of abnormal immature cells in the bone marrow, disrupting normal haematopoietic function [1]. Patients with leukemia often experience symptoms, such as anaemia, bleeding, fever, and tissue and organ infiltration [2,3]. According to the Global Cancer Epidemiology Database (GLOBOCAN) 2020, leukemia ranks 10th in terms of mortality [4]. The two primary types of AL are acute lymphoblastic leukemia (ALL) and acute myeloid leukemia (AML). ALL primarily affects children aged 1–4 years and adults aged ≥55 years, with higher survival rates in children. AML spans all ages and is diagnosed at an average age of approximately 68 years, with survival rates varying from 40% for patients aged <60 years to and 10–20% for those aged ≥60 years. [5,6]. AL’s exact cause of AL is unclear, but it is believed to arise from a complex interplay of multiple factors, including exposure to ionizing radiation, certain chemical agents, genetic predisposition, and aberrant immune responses to infections [7–11]. The immune system is involved in the development, progression, and treatment of leukemia [12].

The gastrointestinal microbiome plays a pivotal role in shaping and maintaining the immune system [13]. Gut microbiota consists of various bacterial species, with Firmicutes and Bacteroidetes being the dominant members [14]. These microbes interact with intestinal epithelial cells, mucosal layer, and intestinal-associated lymphoid tissue to maintain intestinal integrity and immune function [15]. Disruption of the gut microbiota can lead to inflammation and affect the intestinal barrier. Furthermore, the gut microbiota can affect peripheral immune cells and haematopoietic stem cells in the bone marrow [16]. Studies have shown that gut microbiota influences the development and differentiation of myeloid progenitor cells, revealing the significant role of intestinal bacteria in controlling immunity during haematopoiesis [17]. The composition and distribution of the intestinal microbiota can potentially contribute to the development and progression of leukemia, as confirmed in mouse models [18–20]. Furthermore, reduced biodiversity of the microbiota, as well as butyrate, a product of certain gut bacteria, in AML patients has been associated with disease progression in mice and AML patients [21].

Chemotherapy remains the primary treatment option for leukemia; however, it often leads to severe side effects including neutropenia, which increases the risk of infection [22]. To mitigate this risk, patients are often prescribed prophylactic antibiotics during chemotherapy [23]. Antibiotics can also significantly affect the gut microbiota. Research has indicated that antibiotics lead to reduced bacterial diversity, loss of specific bacterial populations, emergence of antibiotic-resistant strains, and upregulation of antibiotic resistance genes (ARGs) [24–26]. Secondary infections caused by antibiotic-resistant bacteria are a major cause of death in patients with leukemia. Overuse of antibiotics has led to antibiotic resistance, a global public health concern [27]. Antibiotic use can promote the selection and spread of ARGs among the intestinal bacteria. This, in turn, increases the risk of ARGs residing in commensal bacteria and subsequently passing on to pathogens [28]. Patients undergoing leukemia treatment, who often have weakened immune systems, are particularly susceptible to these changes. The impact of antibiotic use on the resistance profiles of leukemia and lymphoma patients has been studied, revealing the development of resistance in response to repeated antibiotic courses [29,30]. Metagenomic sequencing of faecal bacterial DNA has the potential to evaluate antibiotic strategies during chemotherapy and to guide the clinical use of antibiotics.

The intricate interplay between gut microbiota, leukemia treatment, and antibiotic resistance forms a complex network of interactions with profound implications for patient outcomes. Gut microbiota, which is pivotal in shaping and maintaining the immune system, undergoes significant alterations during leukemia, potentially influencing disease progression [31]. The proposed study, employing metagenomic sequencing and clinical data analysis, aimed to unravel the dynamics of the gut microbiota and ARGs in patients with AL before and after chemotherapy, particularly during induction chemotherapy. This study provides critical insights into how the composition of the gut microbiota changes in response to treatment, the impact of antibiotic use on microbial diversity, and the development of ARGs. Understanding these intricate relationships may inform the optimization of leukemia treatment strategies, enhance patient safety during chemotherapy, and guide judicious antibiotic use to mitigate resistance risks. While this study provides valuable insights into the complex relationships between gut microbiota, leukemia, chemotherapy, and antibiotic resistance, its findings should be interpreted with caution due to the small sample size. These results contribute to our understanding of these interactions and may inform future research aimed at improving treatment outcomes and patient safety. Further studies with larger cohorts are necessary to validate these findings and explore their potential therapeutic implications.

## Materials and methods

### Study subjects

In this study, we focused on examining changes in gut microbiota during the initial phases of chemotherapy. We included 10 patients with AL who were newly diagnosed at the Second Affiliated Hospital of Shantou University Medical College between May 2021 and May 2022, including five patients with ALL and five patients with AML. The diagnosis of AL is based on morphological, immunological, cytogenetic, and molecular criteria. Chemotherapy was the only treatment option. All patients received conventional induction chemotherapy regimens comprising antineoplastic drugs. However, we did not differentiate between high- and low-sensitivity regimens in our study. Notably, the patients had not undergone any previous rounds of chemotherapy and were, therefore, considered treatment-naïve. Patients with relapsed or refractory AL were excluded from this study. Patients who had received various antibiotics before the study were excluded. This study was reviewed and approved by the Ethics Committee of the Second Affiliated Hospital of the Shantou University Medical College. This study was conducted following the ethical principles outlined in the Declaration of Helsinki.

### Sample collection

Stool samples were collected from patients before (pre) and two weeks after (post-) chemotherapy. The day of induction chemotherapy was defined as Day 0. The period before chemotherapy was defined as the period between admission and the induction of chemotherapy. If the patient had not defaecated or had no preserved stool samples under regulation during this period, a window period of +1 days was allowed. Two weeks after chemotherapy, it was defined as day 14 of the induction chemotherapy. A window period of ±1 days was allowed. The stool sample (0.5 g) was weighed, placed in a clean and dry sampling tube, and stored at −80°C until analysis. The definition of neutropenia fever (NF) is that the patient’s absolute neutrophil count (NEC) ≤ 0.5 × 10^9^/L and a single temperature ≥ 38.0°C sustained over 1 h [32]. Stool samples from patients with neutropenia fever were designated as NF, whereas those from patients without neutropenia fever were designated as the no-neutropenic fever (No NF) group.

There were three NF and seven No NF pateints in pre-chemotherapy group and whereas two NF and eight No NF pateints in post-chemotherapy group.

### Clinical data collection

Patient clinical data including age, sex, body temperature, type, dosage, and duration of antibiotic use were obtained from the hospital’s electronic medical record system. The antibiotics used included β-lactamases, glycopeptides, fluoroquinolones, sulphonamides, glycylcyclins, and macrolides. β-lactamases are further divided into cephalomycin, cephalosporin, penam, and carbapenem. Table S1A shows the antibiotic dosage used for each sample. Antibiotic dosage was calculated as the antibiotic dosage during the period from admission to the day stool samples were collected × the time of use.

### Experimental design, metagenomic sequencing and bioinformatic analyses

DNA was extracted from collected samples using the cetyltrimethylammonium bromide (CTAB) method. An Agilent 5400 instrument (Agilent Technologies, USA) was used to determine the concentration, integrity, and purity of the extracted DNA. DNA samples with OD values between 1.8 and 2.0 were used, and approximately 1 μg of DNA from each sample was used to construct a library. A library was constructed using the NEB Next®Ultra™ DNA Library Prep Kit (Illumina). The qualified DNA samples were randomly fragmented to a size of 350bp bp using a Covaris ultrasonic crusher (Covaris S2 System, United States). The entire DNA library was prepared by terminal repair, poly A tail, sequencing-spliced, purified, and amplified by PCR. Finally, the AMPure XP nucleic acid purification kit (Beckman Coulter, USA) was used to purify the PCR products. An Agilent 5400 instrument was used to determine the insert size of the library. Real-time PCR was used for quantitative analysis of the library concentration. According to the manufacturer’s instructions, index-coding samples were clustered on a cBot Cluster Generation System using the Illumina PE Cluster Kit (Illumina, United States). After cluster formation, the DNA library was sequenced on an Illumina Novaseq 6000 platform, and 150 bp double-ended readings were generated. The bacterial genomic sequences were deposited in the NCBI Sequence Read Archive (accession number: PRJNA1050658) and are freely available.

Metagenomic sequencing was performed using the Illumina NovaSeq high-throughput sequencing platform to obtain raw data in the FastQ format. Raw data were preprocessed using KneadData software (a combination of Trimmomatic and Bowtie2) to obtain high-quality data [33,34]. Considering the possibility of host contamination in the samples, clean data were compared to the host genome and Bowtie2 software [35] was used to filter sequences from the host to obtain valid sequences for subsequent analysis. FastQC was used to test the effectiveness and rationality of the quality control. Kraken2 [36] and a self-built microbial nucleic acid database (screening NCBI NT nucleic acid database and RefSeq whole-genome database belonging to bacterial, fungal, viral, and archaeal sequences) were used to calculate the number of sequences containing species, whereas Bracken [37] was used to predict the actual relative abundance of species in the samples. DIAMOND software [38] was used to compare the quality-controlled and de-host sequences of each sample with the Comprehensive Antibiotics Research Database (CARD,Version 3.2.5, released in September 2022) to obtain ARGs information [39] while filtering out sequences failing the alignment (parameters: -e 0.001 (e-value <1e-3) -i 80 (percent identity > 80%). The clean reads after quality control and de-hosting were blasted to the database (Uniref90) using Humann2 software (based on DIAMOND) [40] and KEGG functional information, according to the correspondence of the Uniref90 ID and KEGG databases.

### Network and statistical analyses

SPSS (version 26.0) was used to compare differences between the two groups. The pair *t*-test was used if the data followed a normal distribution. Otherwise, the Wilcoxon match-pairs signed rank test was used. GraphPad Prism 9.5 was used to draw the box plot. The abundance table was ranked using non-metric multidimensional scaling (NMDS) based on Bray-Curtis distance, and the β-diversity between the two groups was compared using permutation multiple analysis of variance (PERMANOVA). The Venn diagram package in the R software 4.3.0 was used to draw a Venn diagram. The α diversity was calculated using the vegan package. The Psych package was used for Spearman’s correlation analysis, where the P-values were adjusted using the False Discovery Rate (FDR) correction method specifically for the correlations between ARGs, gut bacteria, and antibiotic dosage. Due to the small sample size, comprehensive P-value adjustments across all analyses were not feasible. We clearly state that our study is exploratory and, that not all tests underwent adjustments for multiple testing. This limitation is highlighted to underscore the preliminary nature of our findings. The correlation between ARGs, gut bacteria, and antibiotic dosage was visualized using the interactive platform Gephi 0.10.1. Linear discriminant analysis Effect Size (LEfSe) analysis was performed using the Wilcoxon rank sum test, and the impact on species with significant differences was assessed by Linear Discriminant Analysis (LDA), showing species with differences in horizontal bar charts. This approach reflects a balance aimed at identifying significant trends without the undue loss of data sensitivity, which is particularly crucial given our limited sample size.

## Results

### Clinical characteristics and laboratory parameters

Based on the inclusion and exclusion criteria, 10 patients with AL who received induction chemotherapy were included in this study. Twenty stool samples were collected, including 10 (samples 1–10) in the pre-chemotherapy group and 10 (samples 11–20) in the post-chemotherapy group. The clinical characteristics and detailed laboratory parameters of patients before and after chemotherapy are shown in Table 1.

**Table 1.** Characteristics of the patients under study and laboratory parameters in the pre- and post-chemotherapy groups.

No. of Patients	Sample Name (Pre/Post)	Age	Febrile (F)/Not febrile (N)	Leukemia type	Sex	Survival Outcome	Collection time (day) (Pre/Post)	ADA(U/L) (Pre/Post)	TBA (μmol/L) (Pre/Post)	LDH (U/L) (Pre/Post)	CREA (μmol/L) (Pre/Post)	UREA (mmol/L) (Pre/Post)	WBC (×109/L) (Pre/Post)	NEC (×10^9^/L) (Pre/Post)	NF status	PLT (×109/L) (Pre/Post)	CRP (mg/L) (Pre/Post)	PCT (ng/mL) (Pre/Post)	AST (U/L) (Pre/Post)	ALT (U/L) (Pre/Post)	bone marrow hyperplasia * (Pre/Post)	bone marrow blast cells (%) (Pre/Post)	granulocytes (%) (Post)	erythroid (%) (Post)	mega karyocyte (%) (Post)
1	1/11	11	F/N	ALL	M	L	−3/13	38/10	8.76/27.08	511/NA	69.9/3.1	5.54/9.7	2. 9/2.8	0.78/0.77	No NF/No NF	46/15	5/0.52	0.15/0.31	20/25	7/233	2/2	74/2	8	45.5	6
2	2/12	5	N/N	ALL	F	L	−2/13	17/5	2.75/12.65	385/342	48.7/48	4.01/7.61	11/2.8	1.14/0.57	No NF/No NF	153/140	59.1/0.31	0.1/0.06	19/18	8/36	5/3	83.5/1	4	78.5	25
3	3/13	13	F/F	AML	M	L	−4/14	77/8	1.82/0.95	904/165	195.6/118	11.9/14.19	206.6/1.5	0.00*/0.71	NF/No NF	14/5	15.13/13	3.32/11.33	16/6	11/10	5/5	85/0	79.5	18.5	27
4	4/14	57	F/F	AML	M	D	−2/13	13/13	1.57/4.42	511/315	95.3/79.9	4/6.34	15.8/0.6	0.44/0.03	NF/NF	21/32	31.23/73.9	0.05/0.05	23/21	12/12	4/2	57.5/2	57	NA	10
5	5/15	60	F/F	AML	F	D	−2/14	27/6	2.51/5.22	771/NA	82.5/70.7	4.49/3.95	63.2/4.9	21.74/2.32	No NF/No NF	162/2	7.39/NA	0.05/0.09	19/7	13/12	3/2	75/0	63	5	0
6	6/16	58	F/F	AML	M	D	−5/14	25/6	2.54/9.35	435/89	88.8/89.5	7.18/10.12	42.3/0.3	8.04/0.01	No NF/NF	13/19	55.43/175.77	1.02/2.37	16/10	15/11	4/NA	88/NA	NA	NA	NA
7	7/17	60	F/N	AML	F	L	1/15	10/18	4.29/2.64	202/NA	92/101.5	7.64/9.49	0.6/3.2	0.19/1.56	NF/No NF	47/64	0.89/4.78	NA/NA	17/59	22/68	3/2	60/0	14	33	0
8	8/18	4	F/N	ALL	M	L	1/15	33/13	7.19/4.76	393/NA	47.5/47.5	2.76/10.26	69.6/1.8	65.28/0.04	No NF/No NF	64/74	39.17/2.46	NA/NA	24/14	6/17	4/1	79.5/0	0.5	16	0
9	9/19	7	F/N	ALL	M	L	−1/14	24/7	6.53/22.32	354/145	48/51.3	2.62/5.13	5.6/2.8	3.15/0.7	No NF/No NF	95/95	153/1.78	NA/0.05	18/10	19/16	4/1	68.5/0	11	3	0
10	10/20	7	N/N	ALL	F	L	−1/14	16/9	11/13.92	282/NA	50/57.4	5.56/9.59	20.1/6.9	3.42/1.55	No NF/No NF	19/27	9.81/0.49	0.08/0.27	19/10	10/15	5/5	71.5/50	3.5	6	0

**Pre**: pre-chemotherapy; **Post**: post-chemotherapy; **M**, male; **F**: female; **L**: alive; **D**: dead; **ADA**: Adenosine Deaminase; **TBA**: Total Bile Acid; **LDH**: Lactate Dehydrogenase; **CREA**: Creatinine; **WBC**: White Blood Cell; **NEC**: Neutrophil absolute value; **PLT**: Blood Platelet; **CRP**: C-reactive Protein; **PCT**: Procalcitonin; **AST**: Aspartate Aminotransferase; **ALT**: Alanine Aminotransferase. *Bone marrow hyperplasia: obviously reduced = 1, reduced = 2, active = 3, obviously active = 4, and extremely active = 5 **NF**: Neutropenic fever **No NF**: No Neutropenic fever *: This result was obtained from manual microscopy.

### Diversity and richness analysis of gut microbiota

**Figure 1.**
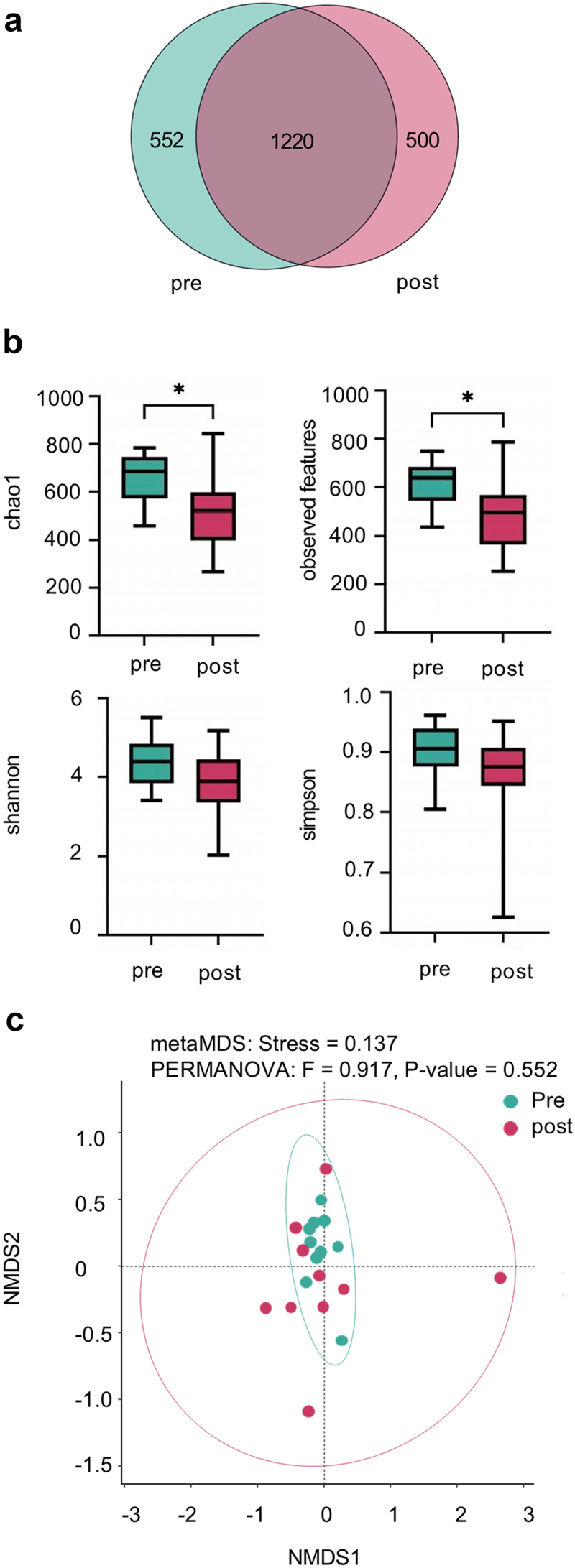
Comprehensive analysis of microbial community dynamics pre- and post-chemotherapy. (a) Venn diagram depicting species-level taxonomy, highlighting shared and unique taxa between the pre-chemotherapy (pre) and post-chemotherapy (post) groups. (b) Box plots showing the comparison of alpha diversity indices (Chao1, observed features, Shannon, Simpson) within the two groups, with paired *t*-tests revealing statistical significance (* denotes *p*<0.05). (c) Non-metric multidimensional scaling (NMDS) with Bray-Curti’s distance to visualize beta diversity, and the resulting PERMANOVA-derived *p* value signifies the overall microbial community dissimilarity between the two groups.

In patients with leukemia pre- and two weeks post-chemotherapy, the number of common and unique operational taxonomic units (OTUs) was determined by drawing a Venn diagram. The number of unique OTUs in the pre-chemotherapy group was 552 and the number of unique OTUs in the post-chemotherapy group was 500, whereas the number of OTUs shared by the two groups was 1220 (Figure 1(a)).

Alpha diversity analyses were performed to assess variations in species richness and evenness between pre- and post-chemotherapy cohorts, utilizing established indicators such as the Chao1 index, observed features, Shannon index, and Simpson index. The results showed a statistically significant reduction in both the Chao1 index and the observed features in the post-chemotherapy group (*p* < 0.05), indicative of diminished species richness compared to the pre-chemotherapy cohort (Figure 1(b)). This reduction suggests a discernible effect of chemotherapy on the overall microbial community composition. Furthermore, the Shannon and Simpson indices were reduced in the post-chemotherapy group, although the observed differences were not statistically significant (*p* > 0.05) (Figure 1(b)). These findings suggest potential changes in microbial richness following chemotherapy, highlighting the importance of further investigating the distribution and balance of microbial taxa within the community, despite the lack of significant shifts in microbial evenness.

Beta diversity algorithms were employed to evaluate microbial community composition across the pre- and post-chemotherapy groups. NMDS plot (Figure 1(c)) provides a visual representation of the microbial community composition in pre- and post-chemotherapy samples. The NMDS stress value of 0.137 indicates a fair representation of the data in two dimensions. PERMANOVA analysis (F = 0.917, P-value = 0.552) revealed no statistically significant difference in overall microbial community composition between pre- and post-chemotherapy groups. The plot shows considerable overlap between the two groups, with both pre-and post-chemotherapy samples distributed across the plot space. While there is no clear separation between groups, we observed a slight trend towards increased dispersion in the post-chemotherapy samples compared to the more tightly clustered pre-chemotherapy samples. One post-chemotherapy sample appears as an outlier, potentially representing individual variation in response to treatment. These observations suggest that while chemotherapy did not induce significant shifts in overall community structure, there may be subtle changes or individual responses that warrant further investigation using more sensitive or targeted analytical approaches.

To address potential confounding factors, we conducted comprehensive analyses of bacterial beta diversity, considering age, sex, and neutrophil count variations among patients. We employed NMDS analysis based on Bray-Curtis distances, coupled with PERMANOVA, as illustrated in Figure S1. Our analyses revealed no statistically significant differences in beta diversity between paediatric and adult patient groups (Figure S1a), male and female cohorts (Figure S1b), or among groups with normal neutrophil counts, neutrophilia, and neutropenia (Figure S1c) (all p > 0.05). These findings suggest that the observed microbial community structures were not significantly influenced by these potential confounding factors, thereby strengthening the robustness of our primary results. However, we acknowledge that other unmeasured variables such as low and high-intensity regimens and cancer type may still play a role in shaping the gut microbiome composition in our study population. This additional analysis provides a more nuanced understanding of the factors influencing gut microbiota in our study cohort and reinforces the validity of our main findings.

### Analysis of types and diversity of gut microbiota resistance genes

**Figure 2.**
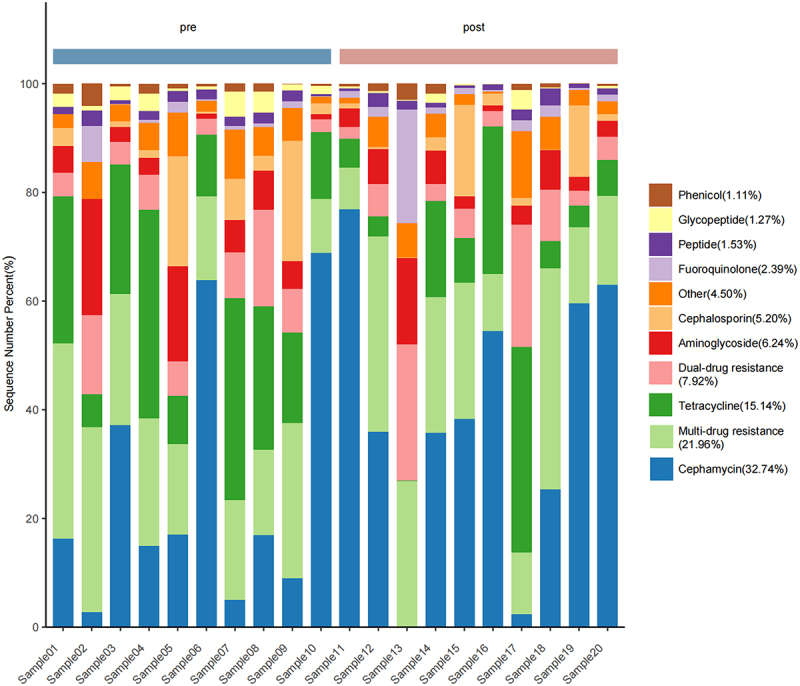
Distribution of antibiotic resistance genes (ARGs) in pre- and post-treatment samples. This stacked bar chart illustrates the relative abundance of various ARGs in individual patient samples before and after treatment. Each bar represents a single sample, with colors corresponding to different ARG categories as indicated in the legend. The x-axis shows individual sample IDs, while the y-axis represents the percentage of each ARG type. The pre-treatment samples are shown on the left half of the chart, and post-treatment samples on the right.

In this study, we explored the identification and prevalence of antibiotic resistance genes (ARGs) in stool samples, focusing on their resistance to various antibiotics. If an ARG was found to confer resistance to two types of antibiotics, it was categorized as a determinant of dual-drug resistance, whereas ARGs conferring resistance to more than two types of antibiotics were categorized to determine multidrug resistance. Notably, ARGs exhibit resistance to a diverse array of antibiotics, with some strains being resistant to as many as 28 antibiotics. The bar chart (Figure 2) and heat map (Figure S2) illustrate the distribution of the ARGs across the samples. Cephamycin was the most prevalent type, constituting 32.74% of the relative abundance. Multidrug resistance-related ARGs followed closely, at 21.96%, whereas tetracyclines, dual-drug resistance, aminoglycosides, and cephalosporins contributed significantly in distinct proportions.

To assess the diversity of ARGs in both the pre-chemotherapy and post-chemotherapy groups, we employed the chao1 index, observed features, Shannon index, and Simpson index to analyse alpha diversity. Upon applying paired t-tests, significant differences emerged in some alpha diversity metrics between the two groups (Figure S3a). The Chao1 index, which estimates species richness, showed a significant increase in the post-chemotherapy group compared to the pre-chemotherapy group (*p* < 0.05). Similarly, the number of observed features, representing the actual count of unique ARGs detected, was significantly higher in the post-chemotherapy samples (*p* < 0.05). These results suggest that chemotherapy may lead to an increase in the variety of ARGs present in the gut microbiome.

Interestingly, while the Shannon index appeared slightly lower in the post-chemotherapy group, this difference did not reach statistical significance. The Simpson index also showed a slight decrease post-chemotherapy, but again, this change was not statistically significant. These findings indicate that while chemotherapy appears to increase the richness of ARGs in the gut microbiome, it may not significantly alter the overall diversity and evenness of these genes. Additionally, beta diversity analysis revealed no significant differences between the ARGs in the two sample groups (*p* > 0.05), as illustrated in Figure S3b. These findings provide valuable insights into the intricate landscape of antibiotic resistance in the studied samples, emphasizing the need for comprehensive strategies to address the multifaceted challenges posed by ARGs. This suggests a complex relationship between chemotherapy treatment and ARGs profile in AL patients.

### Community structure analysis of gut microbiota at different taxonomic levels

In a comprehensive analysis of stool samples, 22 distinct bacterial phyla were identified. Notably, the predominant microbial entity was Bacteroidetes, constituting 60.79% and 62.63% of the microbial community in the pre- and post-chemotherapy groups, respectively. Firmicutes was the second most prevalent phylum, with prevalence rates of 22.40% and 19.34% in the pre-and post-chemotherapy groups, respectively.

**Figure 3.**
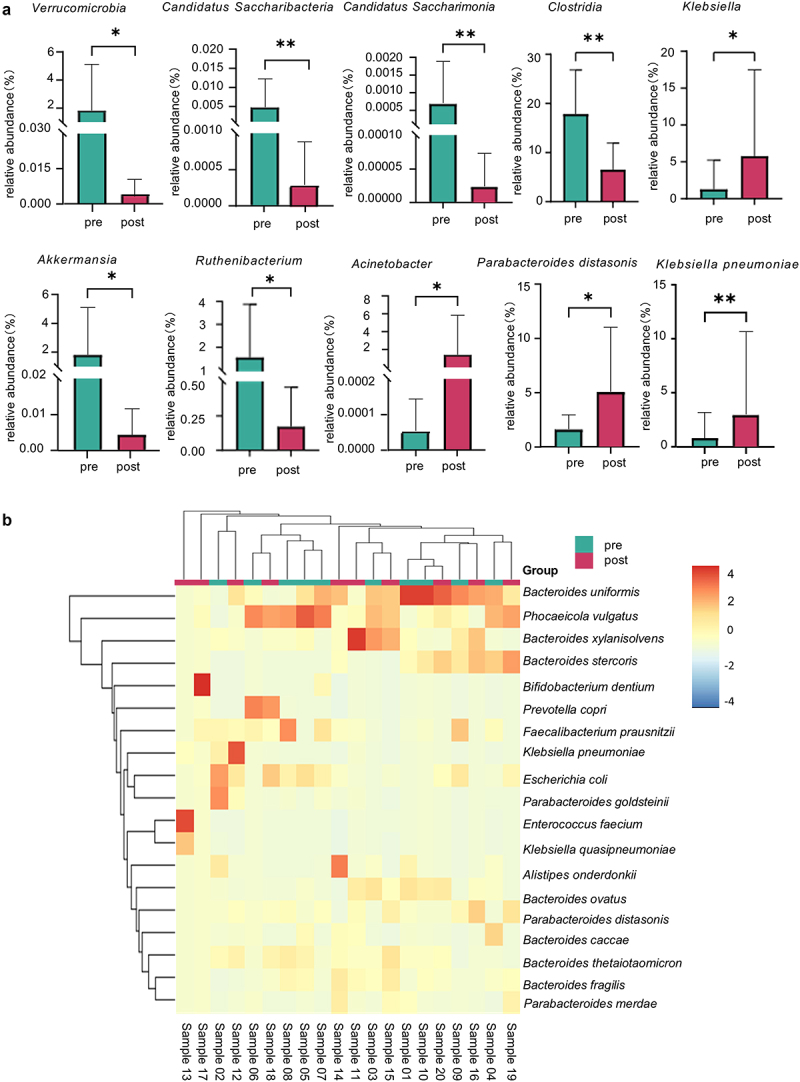
The relative abundance of gut microbiota at different taxonomic levels between pre- and post-chemotherapy groups (a) is the comparison of the relative abundance of bacteria at different taxonomic levels between pre- and post-chemotherapy groups. * denotes the *p*<0.05, ** denotes the *p*<0.01. (b) heat map showing the hierarchical average-linkage clustering of the top 20 bacterial species in each sample based on the Euclidean distance. Each column represents a sample, and each row represents the relative abundance of species in each sample. The color gradients of the squares correspond to different normalized abundance values, ranging from −4 (blue, low abundance) to 4 (red, high abundance), as determined through log transformation. The horizontal bar indicates the grouping of samples, with green representing the pre-chemotherapy group (pre) and red representing the post-chemotherapy group (post).

Further taxonomic refinement revealed distinct microbial diversity comprising 33 classes, 69 orders, 144 families, 413 genera, and 1457 bacterial species. To investigate the differences in bacterial diversity between the pre-and post-chemotherapy groups, the relative abundance of the top 20 bacteria at the phylum, class, genus, and species levels was analysed (Figure 3a). At the phylum level, *Verrucomicrobia* and *Candidatus Saccharibacteria* were significantly less abundant in the post-chemotherapy group than in the pre-chemotherapy group (*p* < 0.05 and *p* < 0.01). We observed a lower relative abundance of *Clostridia* (*p* < 0.01) and *Candidatus Saccharimonia* (*p* < 0.01) at the class level in the post-chemotherapy group. A significant difference in microbial abundance was noted at the genus level for *Klebsiella* and *Acinetobacter*, with a higher abundance in the post-chemotherapy group than in the pre-chemotherapy group. In contrast, the relative abundance of the genus *Akkermansia* and *Ruthenibacterium* was significantly lower in the post-chemotherapy group (*p* < 0.05) than in the pre-chemotherapy group. The relative abundance of *Parabacteroides distasonis* was significantly higher in the post-chemotherapy group (*p* < 0.05). At the species level, *K. pneumoniae* exhibited significantly higher relative abundances in the post-chemotherapy group than in the pre-chemotherapy group (*p* < 0.01).

At the species level, a detailed breakdown identified the top 20 gut microbiota in terms of relative abundance in both the pre- and post-chemotherapy groups (Figure 3(b) and Figure S4). The predominant species identified at the species level were *Bacteroides uniformis, Phocaeicola vulgatus, Bacteroides xylanisolvens, Bacteroides stercoris, Escherichia coli, Parabacteroides distasonis, Bacteroides ovatus, Bacteroides thetaiotaomicron, Enterococcus faecium, Faecalibacterium prausnitzii, fecal Prevotella copri, Bacteroides fragilis, Klebsiella pneumoniae, Alistipes onderdonkii, Parabacteroides goldsteinii, Parabacteroides merdae, Bacteroides caccae, Klebsiella quasipneumoniae*, and *Bifidobacterium dentium*. Notably, *Bacteroides uniformis* exhibited the highest prevalence, constituting 13.44% in the pre-chemotherapy group and 10.43% in the post-chemotherapy group. These results revealed complex changes in the microbial community during chemotherapy, providing insights into how different bacterial species interact in the digestive system.

To further identify the species exhibiting significant distinctions across diverse groups, using the LEfSe method, we stratified the microbiota with significantly disparate abundances between the pre-and post-chemotherapy groups. The magnitude of the LDA value served as a metric for assessing the significance of the differentially abundant species, with a threshold set at LDA > 2 (*p* < 0.05), indicating statistical significance. Notably, the taxa that surpassed the LDA value of 3 were scrutinized.

**Figure 4.**
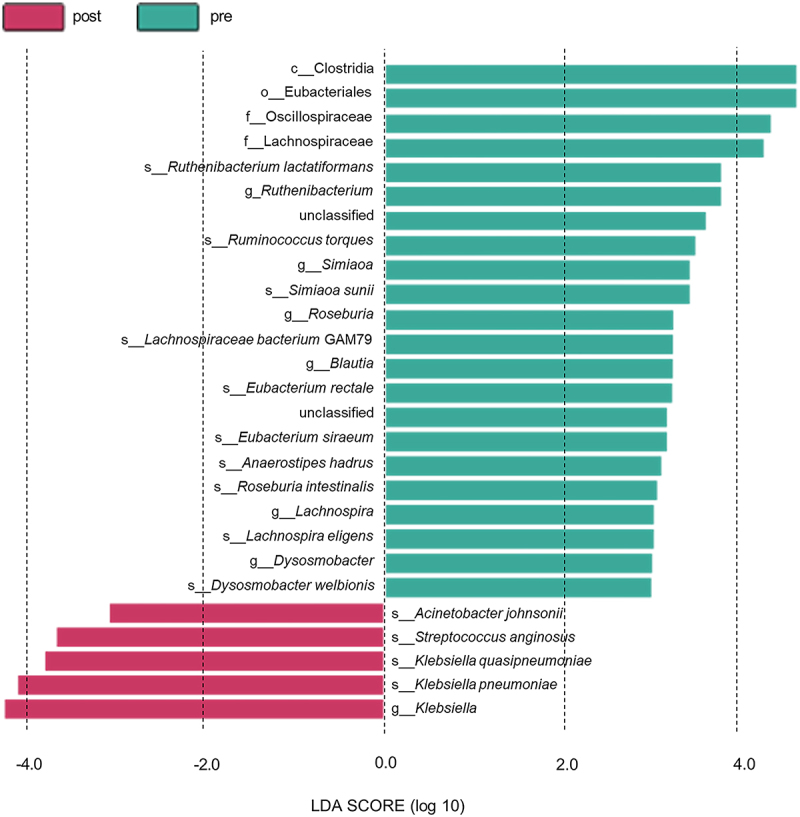
Linear discriminant analyses of the two groups of samples at different levels (LDA>3). In the LDA bar graph, green represents the pre-chemotherapy group (pre), red represents the post-chemotherapy group (post), and the horizontal bar represents the LDA value. The greater the species difference, the higher the LDA value.

Our results revealed a dominance of certain taxa in the pre-chemotherapy group, including *Clostridia, Eubacteriales, Oscillospiraceae, Lachnospiraceae*, *Blautia, Ruthenibacterium, Roseburia, Lachnospira, Dysosmobacter*, and specific species such as *Ruthenibacterium lactatiformans, Ruminococcus torques, Simiaoa sunii*, *Lachnospiraceae bacterium GAM79*, *Eubacterium rectale*, *Eubacterium siraeum*, *Anaerostipes hadrus*, *Roseburia intestinalis*, *Lachnospira eligens*, and *Dysosmobacter welbionis* (*p* < 0.05), as shown in Figure 4 and Figure S5 indicating their higher abundance pre-chemotherapy. Conversely, the post-chemotherapy group exhibited an increased abundance of *Klebsiella*, *Klebsiella pneumoniae, Klebsiella quasipneumoniae*, *Streptococcus anginosus, and Acinetobacter johnsonii* (*p* < 0.05). This comprehensive analysis underscores the differential impact of chemotherapy on specific microbial taxa, with implications for a broader understanding of the dynamics of the gut microbiota in the context of therapeutic interventions.

### Correlation analysis between gut microbiota and blood indicators

**Figure 5.**
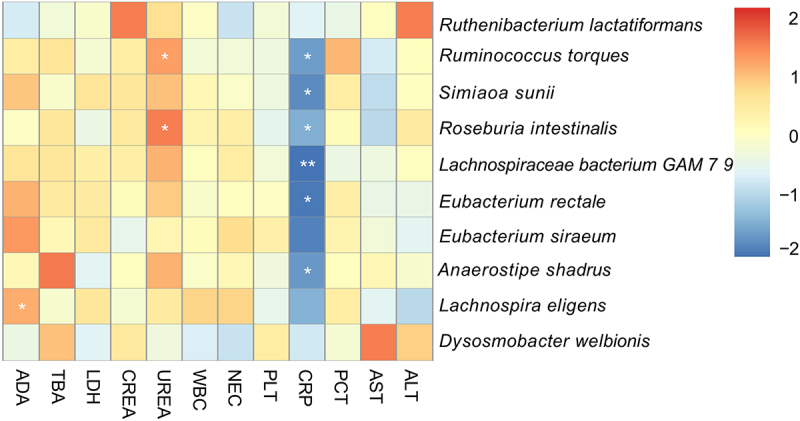
Heat map of Spearman’s correlation between species-level bacteria and blood indicators with LDA>3 in the pre-chemotherapy group. Red represents positive correlation, blue represents negative correlation; the stronger the correlation, the darker the color. * indicates *p*<0.05, ** indicates *p*<0.01.

The correlation between the gut microbiota and key blood indicators in patients was examined using Spearman’s correlation analysis. Figure 5 shows a heatmap of the associations between bacterial species with an LDA > 3 in the pre-chemotherapy group and various physiological parameters, including liver function, kidney function, routine blood tests, inflammatory markers, and additional diagnostic measures. Distinct correlations were observed in the prechemotherapy cohort. Notably, *Ruminococcus torques* (*r* = −0.758, *p* < 0.05), *Simiaoa sunii* (*r* = −0.673, *p* < 0.05), *Roseburia intestinalis* (*r* = −0.661, *p* < 0.05), *Lachnospiraceae bacterium* GAM79 (*r* = −0.806, *p* < 0.01), *Eubacterium rectale* (*r* = −0.745, *p* < 0.05), and *Anaerostipes hadrus* (*r* = −0.758, *p* < 0.05) were negatively correlated with C-reactive protein (CRP). Furthermore, *R. torques* (*r* = 0.648, *p* < 0.05) and *R. intestinalis* (*r* = 0.709, *p* < 0.05) were positively correlated with urea levels. Notably, a positive correlation was found between *Lachnospira eligens* and adenosine deaminase (ADA) (*r* = 0.721, *p* < 0.05). Conversely, no significant correlation was observed between the gut microbial abundance and blood indicators in the post-chemotherapy group.

These findings underscore the intricate relationships between specific gut microbial taxa and key blood parameters, providing valuable insights into the potential influence of gut microbiota on the physiological status of the host, particularly in the pre-chemotherapy setting. The absence of significant correlations in the post-chemotherapy group suggests potential shifts in the gut microbial landscape or altered host-microbe interactions following chemotherapy.

### Correlation analysis between gut microbiome, resistance genes and antibiotic use

**Figure 6.**
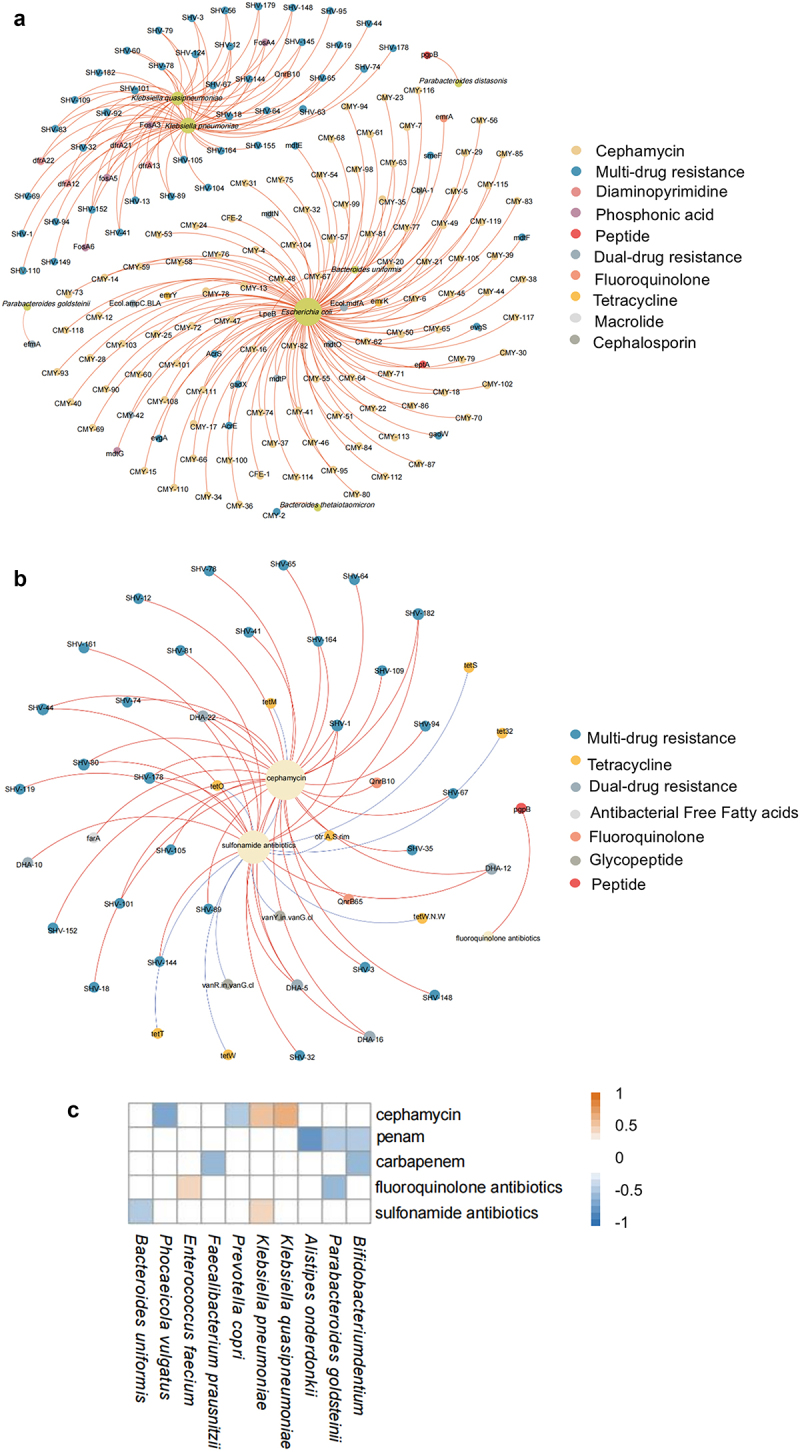
(a) network analysis of co-occurrence patterns between bacterial species and ARGs. The green nodes represent bacterial species. The other nodes are colored according to the ARG type, with each color representing an ARG subtype. The size of each node is proportional to the number of connections. The more connections there are, the larger the node. A connection represented a strong (Spearman’s correlation coefficient *r*>0.8) and significant (*p*< 0.001) correlation. (B) Network analysis of co-occurrence patterns between antibiotics and ARGs. Light-yellow nodes represent the type of antibiotics used. The other nodes are colored according to the ARG type, with each color representing an ARG subtype. The size of each node is proportional to the number of connections. The more connections there are, the larger the node. A connection represented a moderate (Spearman’s correlation coefficient *r*>0.6) and significant (*p*< 0.05) correlation, with 10 blue edges indicating a negative correlation and 46 red edges indicating a positive correlation. (C) Spearman’s correlation heatmap showing the association between the top 20 species and antibiotic dosage from the samples both pre and post-chemotherapy. All the analysis includes the samples both pre and post-chemotherapy. Only bacterial species correlated with the dosage of antibiotics are presented (*p*< 0.05). Orange represents a positive correlation and blue represents a negative correlation, while the degree of correlation is proportional to the color depth. White indicates no correlation.

Spearman’s correlation analysis was performed to determine the correlation between bacterial species and ARGs in faecal samples, and co-occurrence patterns between the two were determined using network analysis. A total of 797 ARGs with high abundance (i.e. at least 40% of the samples identified with ARG) were screened out. The top 20 bacteria with species-level abundance were analysed for their correlation with ARGs. The correlation coefficients were set to *r* > 0.8, and *p* < 0.001 was used to screen for significantly correlated microbial groups and ARGs. A co-occurrence network consisting of 179 nodes (including seven species of bacteria and 172 ARGs) and 206 edges was constructed. As shown in Figure 6(a) and Table S1B, *Escherichia coli* was the potential host for 118 ARGs, *K. pneumoniae* was the potential host for 48 ARGs, and *K. quasipneumoniae* was the potential host for 35 ARGs. *Bacteroides uniformis* was linked to CblA-1 and LpeB, whereas *Parabacteroides distasonis* was linked to pgpB. *Bacteroides thetaiotaomicron* was linked to CMY-2 and *Parabacteroides goldsteinii* was linked to efmA.

The correlation between the antibiotic dosage and ARGs was calculated, and the results were presented using a co-occurrence network composed of 50 nodes (including three antibiotics and 47 ARGs) and 50 edges. As shown in Figure 6(b) and Table S2, the dosage of cephamycin was related to 31 ARGs, sulphonamide antibiotics were related to 24 ARGs, and fluoroquinolone antibiotics were correlated to one ARG (*r* > 0.6, *p* < 0.05).

To explore whether the dosage of antibiotics had an impact on the gut microbiota, we calculated and drew a Spearman correlation heat map between the abundance of the top 20 species-level species and the antibiotic dosage. As shown in Figure 6(c), the dosage of cephamycin was positively correlated with *Klebsiella pneumoniae* (r*=*0.583, p < 0.01) and *K. quasipneumoniae* (r = 0.615, p < 0.05) and negatively correlated with *Phocaeicola vulgatus* (r = 0.-617, p < 0.01) and *Prevotella copri* (r = 0.-490, p < 0.05). Penicillane (Penam) dosage was negatively correlated with *Alistipes onderdonkii*, (r = −0.739, p < 0.001), *Parabacteroides goldsteinii* (r = −0.462, p < 0.05), and *Bifidobacterium dentium* (r = 0.-445, p < 0.05). Carbapenem was negatively correlated with *Faecalibacterium prausnitzii* (r = −0.522, p < 0.05) and *Bifidobacterium dentium* (r = −0.571, p < 0.01). Fluoroquinolone antibiotics were negatively correlated with *Parabacteroides goldsteinii* (r = −0.548, p < 0.05) and positively correlated with *Enterococcus faecium* (r = 0.492, p < 0.05). Sulfonamide antibiotics were negatively correlated with *Bacteroides uniformis* (r = −0.474, p < 0.05) and positively correlated with *K. pneumoniae* (r = 0.496, p < 0.05).

### Analysis of gut microbiota in patients with and without neutropenic fever caused by chemotherapy

**Figure 7.**
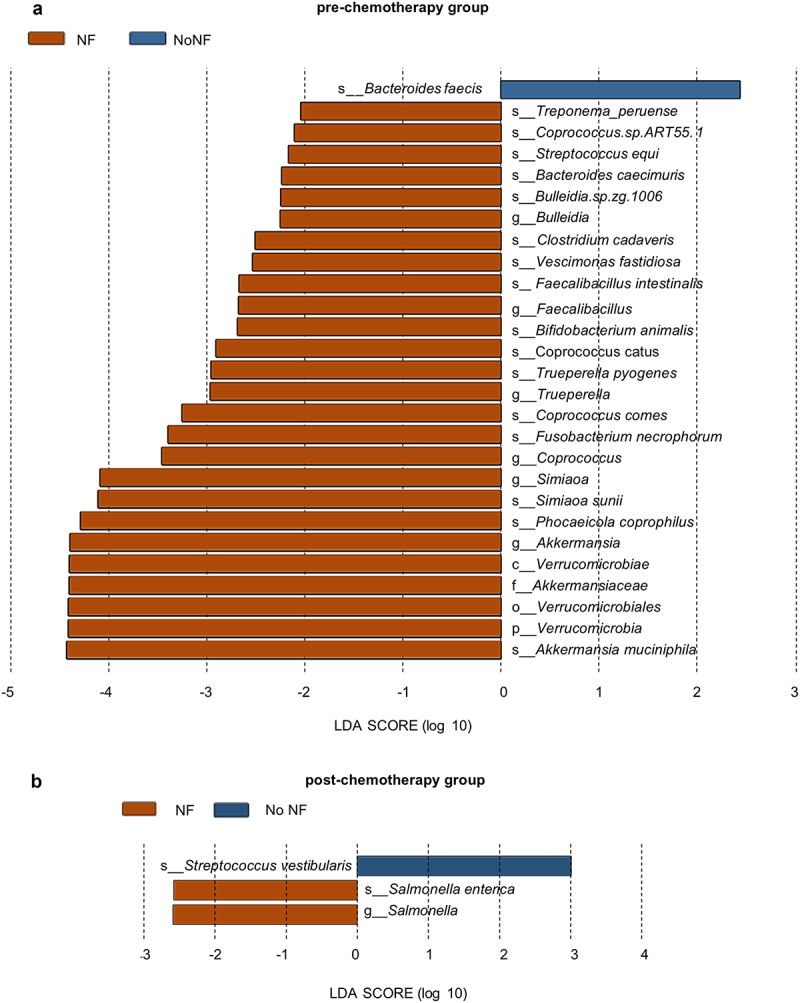
Differential abundance of gut microbiota in patients with and without neutropenic fever. (a) linear discriminant analysis (LDA) effect size (LEfSe) results comparing gut microbiota abundance between patients with neutropenic fever (NF) and without fever (NoNF) before chemotherapy initiation. Orange bars represent taxa enriched in the NF group, while blue bars indicate taxa enriched in the NoNF group. The length of each bar corresponds to the LDA score (log 10), with higher scores indicating greater differential abundance (LDA > 2, *p* < 0.05). (b) LEfSe analysis results comparing gut microbiota abundance between NF and NoNF patients two weeks post-chemotherapy. Color scheme and bar representation are consistent with panel A. Only taxa meeting the significance threshold (LDA > 2, *p* < 0.05) are shown. In both panels, taxonomic levels are indicated by prefixes (s_: species, g_: genus, f_: family, o_: order, c_: class). The x-axis represents the LDA score, reflecting the magnitude of differential abundance between groups.

We stratified the samples from the pre-chemotherapy cohort into Neutropenic Fever (NF) and Non-Neutropenic Fever (No NF) groups based on the occurrence of neutropenic fever at the time of sample collection. Using LDA, coupled with effect size measurements (LEfSe), we identified differentially abundant gut microbiota between the two groups (LDA >2). In the pre-chemotherapy group, a diverse array of microorganisms showed significantly higher relative abundances in the NF group (Figure 7(a)) (*p* < 0.05). These included members of the phylum *Verrucomicrobia* (specifically *Akkermansia muciniphila*), various Firmicutes (including species of *Coprococcus, Faecalibacillus*, and *Clostridium*), and representatives from other phyla such as *Bacteroidetes* (*Phocaeicola coprophilus*) and *Fusobacteria* (*Fusobacterium necrophorum*). Conversely, only *Bacteroides faecis* demonstrated a significantly higher abundance in the No NF group (*p* < 0.05).

For the post-chemotherapy cohort, we employed a similar stratification approach. The NF group showed a predominance of *Salmonella* species, particularly *Salmonella enterica*, while *Streptococcus vestibularis* was more abundant in the No NF group (Figure 7(b)) (*p* < 0.05). These findings suggest potential microbial signatures that may be predictive of neutropenic fever development. The identified taxa could serve as potential biomarkers for risk stratification before chemotherapy initiation. Additionally, the observed differences in microbial composition post-chemotherapy may indicate shifts in the gut microbiome associated with neutropenic fever during treatment.

The distinct microbial profiles observed in No NF patients, both pre-and post-chemotherapy, hint at potentially protective microbial communities. These insights could inform future strategies for modulating the gut microbiome to mitigate the risk of neutropenic fever in patients undergoing chemotherapy.

### Functional analysis of gut microbiota before and after chemotherapy

**Figure 8.**
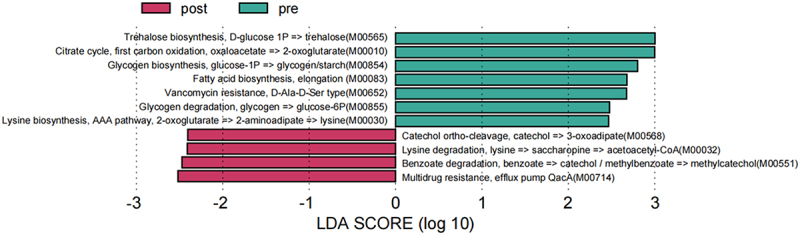
KEGG module for the linear discriminant (LEfSe) analysis of pre- and post-chemotherapy groups (LDA>2). Green represents the pre-chemotherapy group (pre), and red represents the post-chemotherapy group (post). The length of the horizontal bar represents the LDA value. The greater the species difference, the higher the LDA value.

In our investigation of functional changes within intestinal microorganisms, we employed KEGG module annotation to discern chemotherapy-induced alterations. Using the LEfSe method, we identified KEGG modules that exhibited significant differences (LDA >2) between the pre-and post-chemotherapy groups (Figure 8).

In the pre-chemotherapy group, several enriched KEGG modules indicated distinct metabolic adaptations. Notably, the M00565 module, which is involved in trehalose biosynthesis from d-glucose (1P), exhibited increased activity. Trehalose, a known stress response molecule, may be involved in adaptive microbial mechanisms. Additionally, modules associated with carbohydrate metabolism (M00010, citrate cycle) and lipid metabolism (M00854 and M00855, glycogen biosynthesis and degradation) were significantly enriched, suggesting alterations in energy storage and utilization pathways. Furthermore, the M00083 module, which is associated with elongation of fatty acid biosynthesis, displayed enhanced activity, indicating a potential shift in lipid metabolism. Amino acid metabolism was also implicated, as evidenced by the enrichment of M00030 (lysine biosynthesis and AAA pathway) and M00652 (drug resistance, vancomycin resistance, and the D-Ala-D-Ser type).

Distinct KEGG modules were significantly enriched in the post-chemotherapy group. Notably, the M00714 module, which is associated with drug resistance (multidrug resistance and efflux pump QacA), was highly enriched, suggesting a response to the chemotherapeutic agents. Modules related to xenobiotic degradation (M00551, paraben degradation) and amino acid metabolism (M00568, ortho-cleavage of catechol; M00032, lysine degradation) were also enriched, indicating potential alterations in microbial response to xenobiotics and amino acid metabolism.

These findings highlight the dynamic nature of intestinal microbiota in response to chemotherapy, with implications for drug resistance, metabolic adaptation, and xenobiotic degradation. The identification of these enriched KEGG modules provides a foundation for understanding the functional changes within the microbial community and their potential impact on host health during and after chemotherapy. Figure 8 shows the differential enrichment patterns observed in the pre- and post-chemotherapy groups.

## Discussion

The human gastrointestinal microbiota, comprising 40 trillion microorganisms, profoundly influences overall health and extends beyond the gastrointestinal tract [41]. Currently, chemotherapy remains the main treatment for leukemia, although it adversely affects the gut microbiota by altering the microbial abundance, compromising the intestinal barrier, and causing dysbiosis [22,42]. Research on the gut microbiota during chemotherapy has been constrained by a predominant reliance on 16S rRNA sequencing methodologies [6,20]. In our study, we employed metagenomic sequencing, allowing for comprehensive annotation of the microbial community at the genus or species level, and providing a more nuanced understanding of microbial richness during chemotherapy. Our research has revealed a nuanced interplay between chemotherapy, the gut microbiota, and antibiotic resistance. However, it is essential to acknowledge that antibiotic resistance typically evolves over prolonged periods and is influenced by various factors such as antibiotic usage, genetic predisposition, environmental influences, and microbial interactions [43,44]. Although rapid changes in resistance profiles are conceivable, they are more commonly observed over extended durations than within short timeframes [45,46].

The present study showed that bacterial diversity decreased after chemotherapy, suggesting alterations in microbial richness, consistent with the results of previous studies [47–49]. Beta diversity displayed non-significant differences, but increased sample dispersion post-chemotherapy indicated changes in the microbial richness. Chemotherapy drugs can damage epithelial cells and disrupt the intestinal barrier, thereby affecting the gut microbiota and leading to microbial imbalances [42]. The abundance of Phylum *Saccharibacteria*, genera *Saccharimonia*, and *Ruthenibacterium* in the intestine was reduced after chemotherapy, indicating potential chemotherapy sensitivity. Species of the phylum *Candidatus Saccharibacteria* have been implicated in inflammatory bowel disease, bacterial vaginosis, periodontitis, and other inflammatory conditions [50]. *Candidatus Saccharimonas* is often an indicator of poor intestinal microbiota health [51,52]. After two weeks of chemotherapy, there was a significant increase in the abundance of *Klebsiella*, including *K. pneumoniae* and *K. quasipneumoniae*, as well as *Streptococcus anginosus* and *Acinetobacter johnsonii*, which are common pathogenic bacteria. This abundance, along with a decrease in certain beneficial bacteria such as *Lachnospiraceae* family members (e.g. *Roseburia, Lachnospira*, and *Blautia*), suggests substantial intestinal dysbiosis. Reductions in the richness of bacteria, which are responsible for producing butyrate, a key component for maintaining the intestinal barrier, may compromise barrier function, potentially impacting leukemia remission. [21,53]. Further research is required to validate interventions such as butyrate supplementation, maintenance of balance in the intestinal microbiota, and particularly the preservation of beneficial bacteria, which may improve clinical outcomes in patients with leukemia undergoing chemotherapy.

Blood biomarkers may reflect changes in gut microbiota linked to systemic treatment effects [54]. In the current study, some species with significant differences identified by LEfSe were correlated with liver/kidney function and inflammatory indicators. Notably, before chemotherapy, C-reactive protein (CRP) was negatively correlated with short-chain fatty acid producers (*R. intestinalis*, *L. bacterium* GAM79, *Eubacterium rectale, and Anaerostipes hadrus*). These bacteria play crucial roles in anti-inflammatory processes by stimulating cytokine secretion, promoting regulatory T cell differentiation, activating innate lymphocytes, and inhibiting pro-inflammatory cytokines [55–57]. This negative correlation suggests a potential link between the reduced abundance of short-chain fatty acid producers, increased cytokine release, and elevated CRP levels. Additionally, *Ruminococcus torques*, which are known for their adaptation to the intestinal mucus layer [58], were negatively correlated with CRP levels, indicating their potential anti-inflammatory role. In influenza-like illnesses, *Ruminococcus* abundance correlates with elevated *E. coli* levels and increased local/systemic CRP concentrations [59]. We identified a positive correlation between *R. torques*, *R. intestinalis* abundance, and serum UREA concentration.UREA, the main source of nitrogen for colonic endosymbiotic bacteria, may indirectly affect the gut bacterial abundance [60]. The combination of UREA and CREA is usually used clinically to determine whether renal function in patients is impaired while only one case in our cohort observed both elevated CREA and UREA before chemotherapy, indicating renal function impairment. It would be valuable to include a larger cohort of patients with renal impairment induced by chemotherapy to explore whether changes in gut microbiota will affect renal function. Additionally, a positive correlation was identified between *L. eligens* abundance and serum ADA concentration, assessing liver function and potentially influencing tumour development through adenosine degradation [61]. The impact of intestinal microbiota on haematopoietic cells was explored, revealing negative correlations between *Roseburia, Faecalibacterium*, and peripheral blood leukocyte counts in patients with AML [21]. Future studies should expand the cohort to include bone marrow parameters, and provide comprehensive insights into the effects of chemotherapy on bone marrow function.

Empiric antibacterial treatment is crucial for AL patients with compromised immunity, who often require broad-spectrum antibiotics, contributing to the proliferation of antibiotic-resistant microorganisms [29,62]. In our study, *Enterobacteriaceae* and *Enterococcus*, *E. coli, K. pneumoniae, E. faecalis, and E. faecium*, emerged as the most prevalent multidrug-resistant nosocomial pathogens. It has been previously reported that *E. coli, K. pneumoniae, and K. quasipneumoniae* are the top carriers of ARGs in the intestine [63]. Our findings indicate that *E. coli* harbours 118 ARGs, predominantly of the CMY type, conferring resistance to cephamycin within the widely used β-lactam class. β-lactamases, which are commonly responsible for lactam drug resistance, particularly AmpC β-lactamases, play a crucial role in cephalosporin and cephamycin resistance [64,65]. Despite this, there was no correlation between the antibiotic dose and *E. coli* or its associated ARGs. This suggests that β-lactam resistance expressed by *E. coli* in patients with leukemia may not be linked to the antibiotic dosage during leukemia treatment. *K. pneumoniae* potentially hosts 48 ARGs, conferring multidrug resistance with positive correlations with cephamycin and sulphonamide dosages. *K. quasipneumoniae*, which hosts 35 ARGs, overlapped with *K. pneumoniae*-related ARGs. Cumulative cephamycin exposure may contribute to the development of multidrug-resistant ARG, suggesting potential colonization by *K. quasipneumoniae*. Sulphonamide antibiotics, known for broad-spectrum activity [66], showed a positive correlation with *K. pneumoniae* abundance but lacked changes in sulphonamide-related ARGs in our study. Despite complex factors, such as chemotherapeutic toxicity, immune-compromised health status, and dietary changes, our analysis suggests potential links between antibiotic dosage and multidrug-resistant ARGs. Understanding the impact of the evolution of antibiotic resistance on other drugs requires further exploration of cross-resistance mechanisms.

Neutropenic fever (NF) is a common, high-risk complication for AL patients [25,67]. *Akkermansia muciniphila* is a mucin-degrading bacterium with potential probiotic properties that maintain intestinal barrier function [68,69]. However, its excess colonization can disrupt the epithelial barrier and induce inflammation [70]. A previous study found that higher *A. muciniphila* levels predicted higher neutropenic fever risk in acute leukemia patients, potentially by modulating the mucosal interface and synergizing with chemotherapy-induced damage to enhance the absorption of dysbiotic metabolites (Rashidi et al. [71]2021b). In this cohort, higher levels of *A. muciniphila* before chemotherapy in NF group may be a bacterial biomarker for predicting neutropenic fever onset. Interestingly, *Akkermansia* abundance decreased post-chemotherapy, with no difference between NF and No NF groups. Increased abundance of *Salmonella enterica* was found in the NF group after chemotherapy, although our laboratory tests did not indicate that patients were infected with *Salmonella* [72–74]. These findings highlight the unhealthy intestinal state of neutropenic fever patients during chemotherapy. Detecting these microbiota components before or early in chemotherapy could help to assess the risk of infection, guide preventive strategies, and enhance chemotherapy outcomes.

Metagenomic KEGG module analysis revealed disruptions in key metabolic pathways after chemotherapy, indicating potential targets for managing side effects and predicting prognosis. We showed that carbohydrate metabolism pathways, including trehalose biosynthesis, and amino acid metabolism pathways, including lysine biosynthesis, were affected. Trehalose, which is vital for microbiota growth, is utilized by beneficial bacteria, such as *Bifidobacteria*. The impact of chemotherapy on trehalose biosynthesis may decrease the proliferation of beneficial intestinal microbiota, potentially worsening intestinal health [75]. The lysine biosynthetic pathway, which is linked to butyrate synthesis, is crucial for maintaining the intestinal barrier [76] and can be disturbed post-chemotherapy, negatively affecting intestinal health. The post-chemotherapy group exhibited enrichment in the multidrug resistance-related efflux pump QacA pathway. QacA, a multidrug transporter, confers resistance to various antibiotics in *Staphylococcus aureus* [77]. Although *S. aureus* invasion was not observed between the pre- and post-chemotherapy groups, monitoring multidrug-resistant *S. aureus* throughout chemotherapy is essential to understand the potential changes over time.

Overall, our study highlighted significant shifts in microbial diversity during chemotherapy, which affected key microbial richness and function. The correlation between gut microbiota, blood indicators, and ARGs provides crucial insights into the systemic effects of treatment. The identification of specific bacterial species associated with NF highlights the potential biomarkers of infection risk. Understanding these dynamics will pave the way for personalized therapeutic interventions, optimizing leukemia treatment, minimizing antibiotic resistance risks, and ultimately, enhancing patient outcomes and safety during chemotherapy.

## Supplementary Material

Figure S1.tif

Revised_Table_S1 _ clean copy.xlsx

Figure S2.tif

Figure S3.tif

Figure S4.tif

Table S2.xlsx

Figure S5.tif

## Data Availability

Bacterial genomic sequences were deposited in the NCBI Sequence Read Archive (accession number: PRJNA1050658).
