# Supplementary figures and images for: Exploring the dynamics of gut microbiota, antibiotic resistance, and chemotherapy impact in acute leukemia patients: A comprehensive metagenomic analysis
Source: Virulence. 2024 Dec 2;15(1):2428843. doi: 10.1080/21505594.2024.2428843 (PMC11622590; doi:10.1080/21505594.2024.2428843)

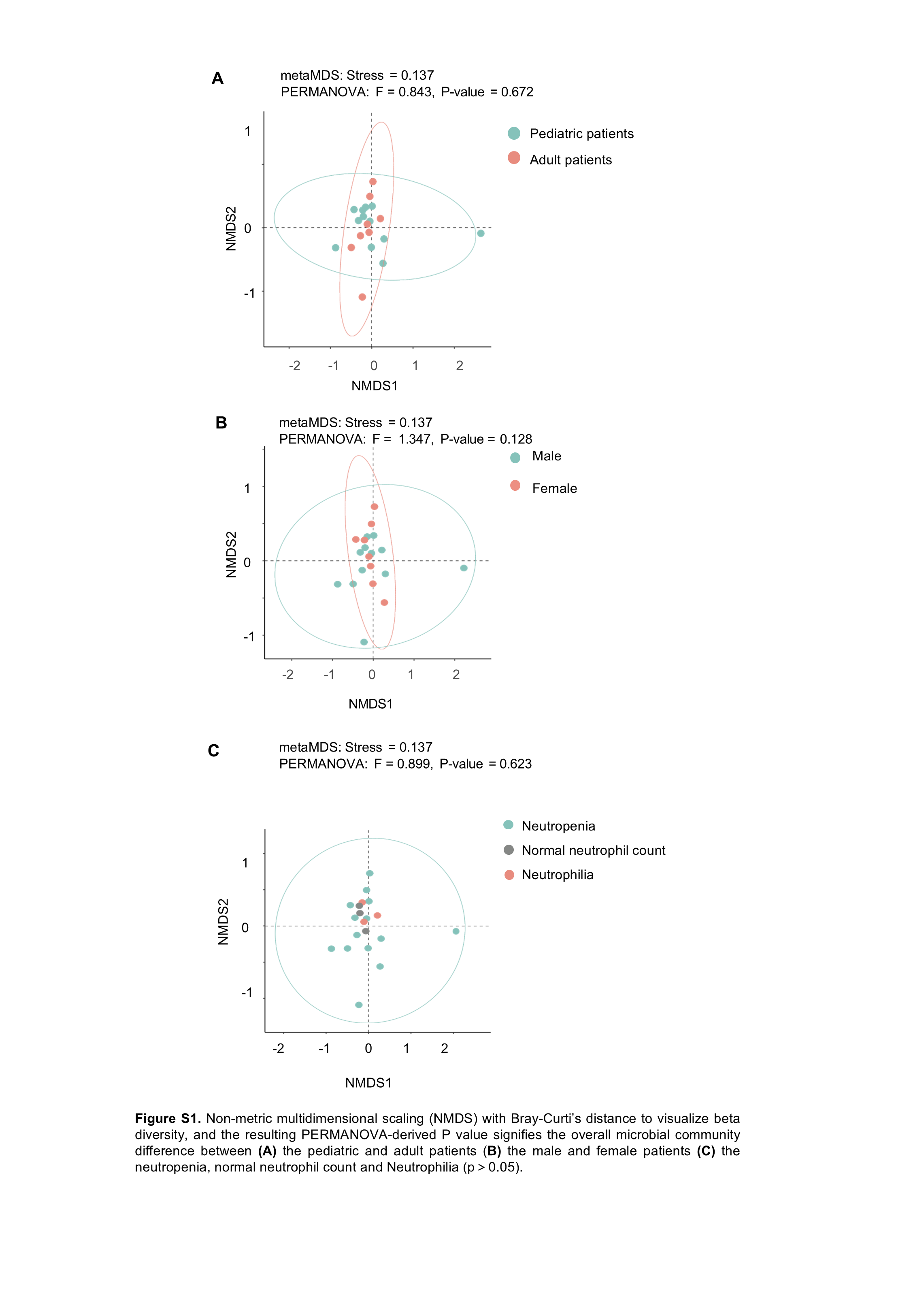

Supplement: Figure S1.tif [file KVIR_A_2428843_SM0733.tif]

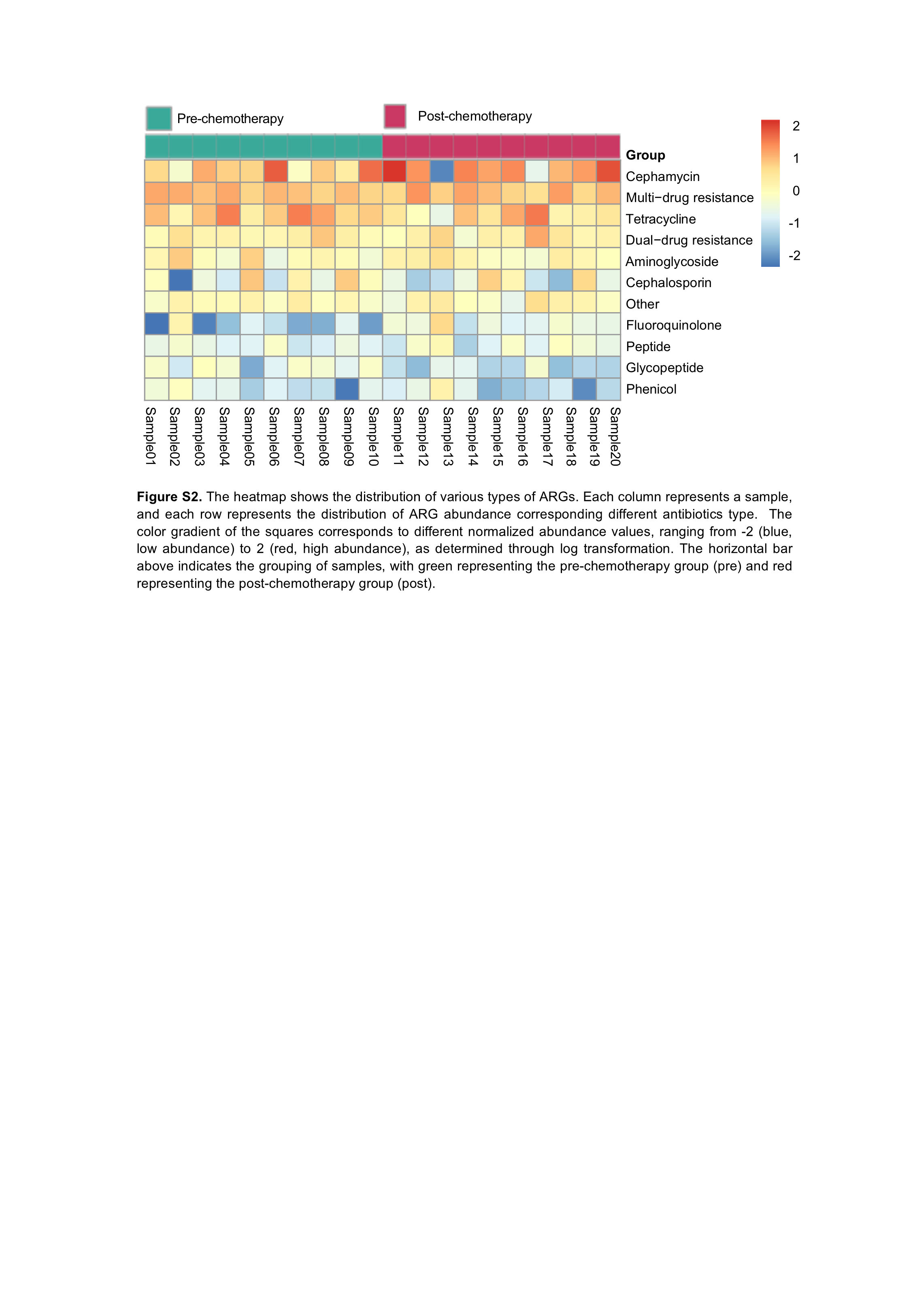

Supplement: Figure S2.tif [file KVIR_A_2428843_SM0731.tif]

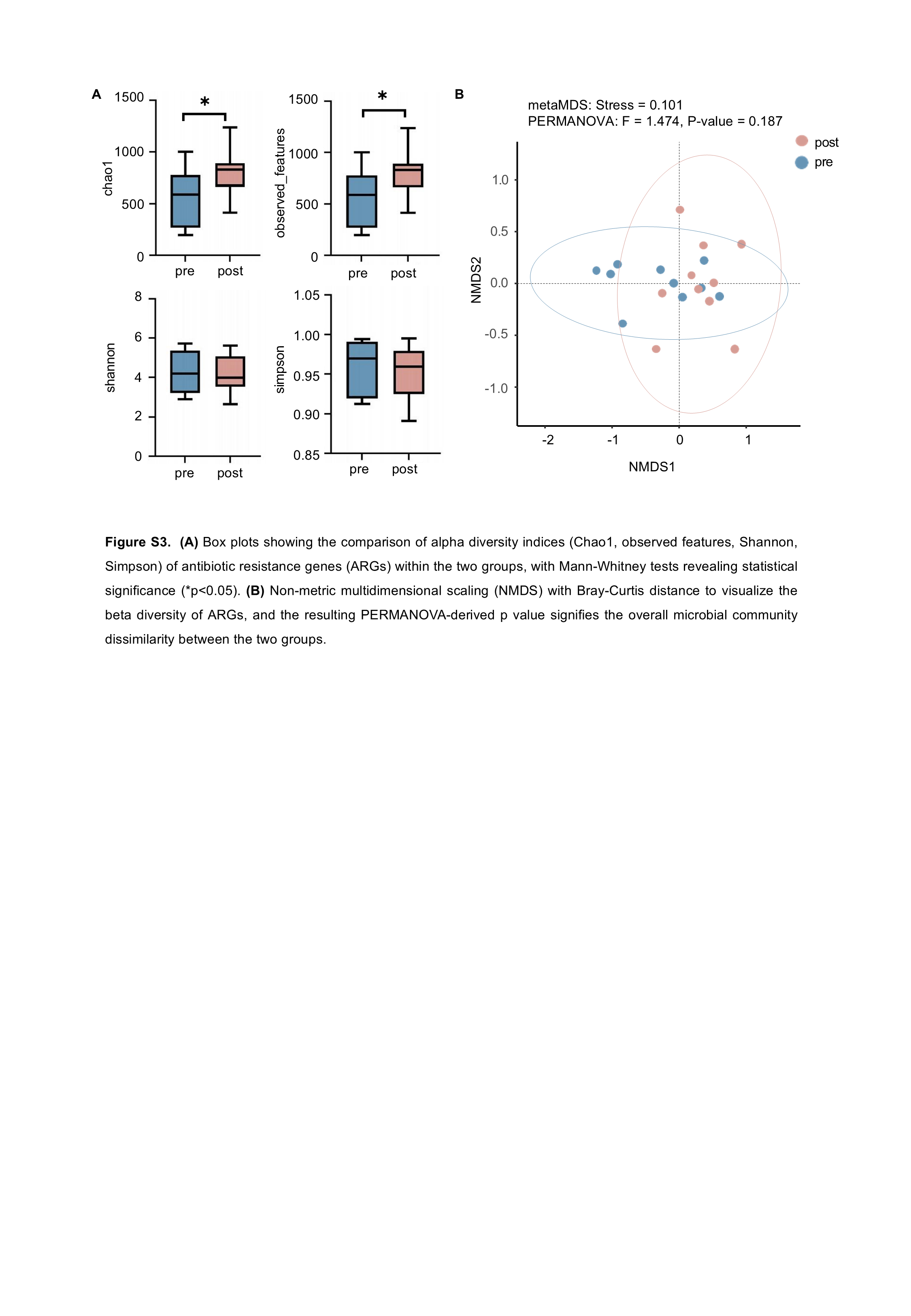

Supplement: Figure S3.tif [file KVIR_A_2428843_SM0730.tif]

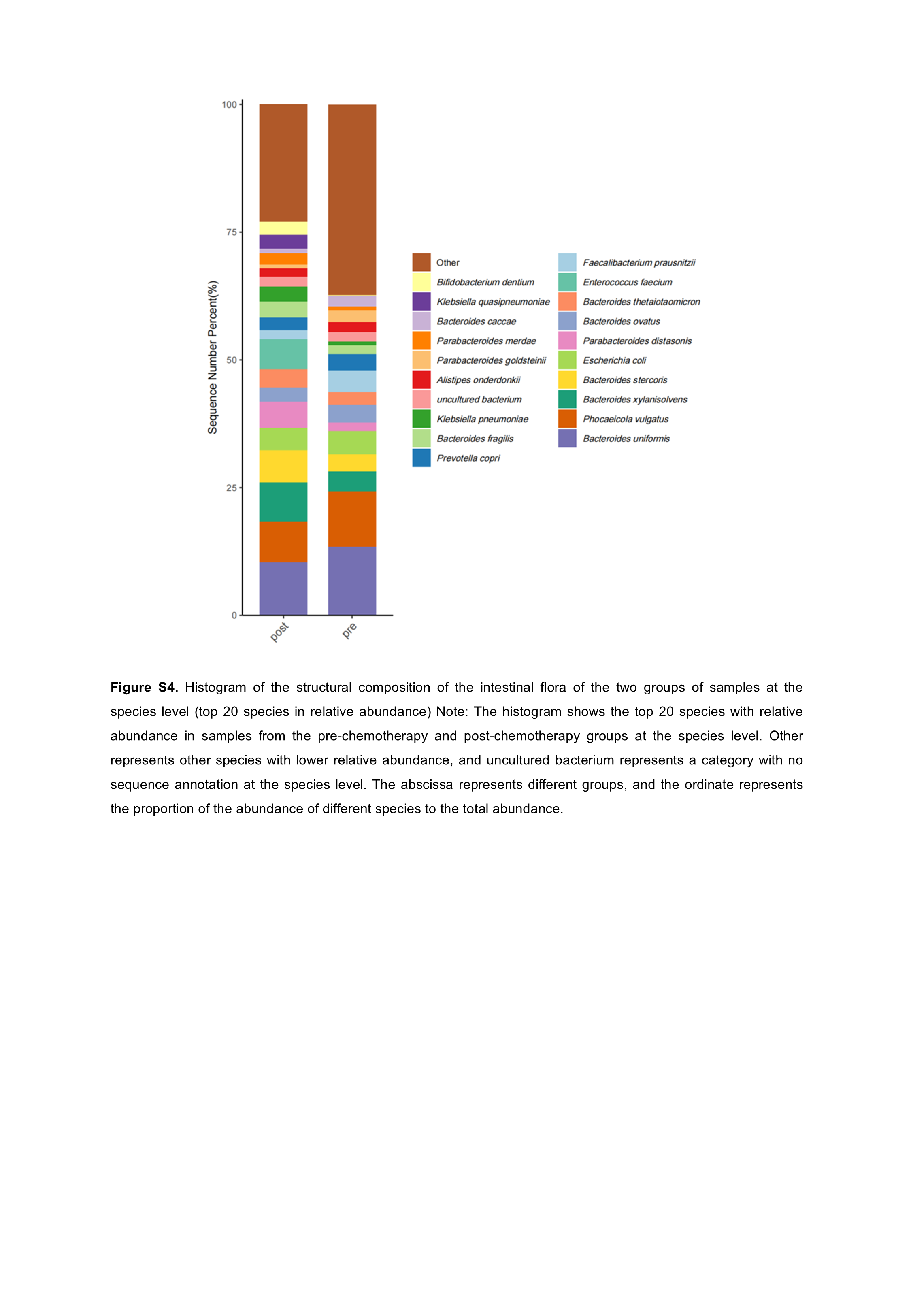

Supplement: Figure S4.tif [file KVIR_A_2428843_SM0729.tif]

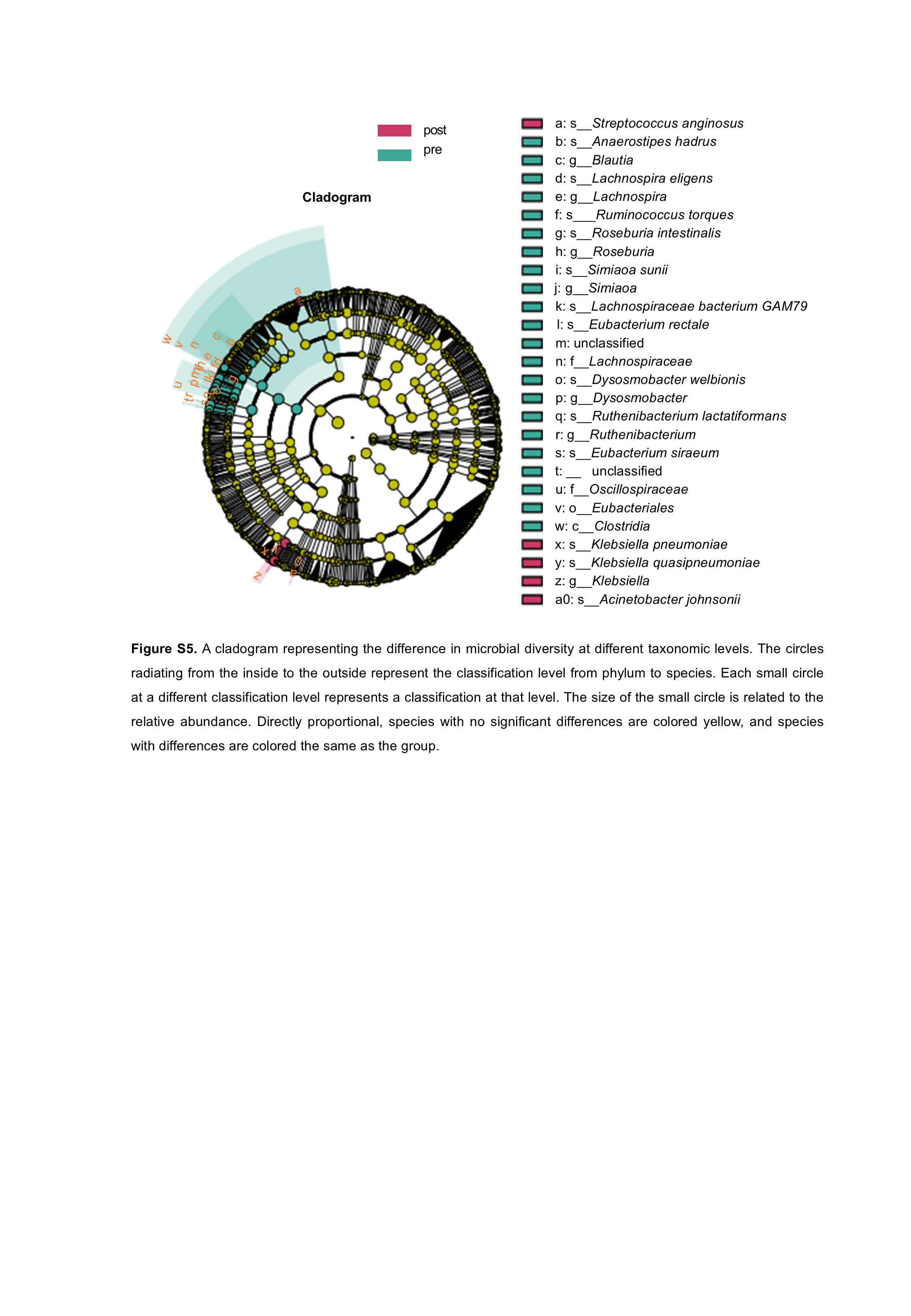

Supplement: Figure S5.tif [file KVIR_A_2428843_SM0727.tif]
